# Retroclavicular approach vs infraclavicular approach for plexic bloc anesthesia of the upper limb: study protocol randomized controlled trial

**DOI:** 10.1186/s13063-017-2086-1

**Published:** 2017-07-21

**Authors:** PL Langlois, AF Gil-Blanco, D Jessop, Y Sansoucy, F D’Aragon, N Albert, P Echave

**Affiliations:** 1 Department of Anesthesiology, Medicine and Health Sciences Faculty, Centre Hospitalier Universitaire de Sherbrooke (CHUS), 3001, 12e Avenue Nord, Sherbrooke, J1H5N4 QC Canada; 2 Department of Anesthesiology, Medicine and Health Sciences Faculty, Centre Hospitalier Universitaire de Laval (CHUL), Quebec, QC Canada

**Keywords:** Regional anesthesia, Brachial plexus anesthesia, Coracoid approach, Retroclavicular approach

## Abstract

**Background:**

The coracoid approach is recognized as the simplest approach to perform brachial plexus anaesthesia, but needle visualization needs to be improved. With a different needle entry point, the retroclavicular approach confers a perpendicular angle between the ultrasound and the needle, which theoretically enhances needle visualization. This trial compares these two techniques. The leading hypothesis is that the retroclavicular approach is comparable to the infraclavicular coracoid approach in general aspects, but needle visualization is better with this novel approach.

**Methods:**

We designed a multicentre, randomized, non-inferiority trial. Patients eligible for the study are older than 18 years, able to consent, will undergo urgent or elective upper limb surgery distal to the elbow and are classified with American Society of Anaesthesiologists risk score (ASA) I-III. They will be excluded if they meet contraindicated criteria to regional anaesthesia, have affected anatomy of the clavicle or are pregnant. Randomization will be done by a computer-generated randomization schedule stratified for each site and in 1:1 ratio, and concealment will be maintained with opaque, sealed envelopes in a locked office. The primary outcome, the performance time, will be analyzed using non-inferiority analysis while secondary outcomes will be analyzed with superiority analysis. Needle visualization will be ranked on a Likert scale of 1–5 that is subjective and represents a pitfall. Two separate persons will rank needle visualization to compensate this pitfall. According to previous studies, 49 patients per group are required for statistical power of 0.90 and one-sided type I error of 0.05.

**Discussion:**

The conduct of this study will bring clear answers to our questions and, if our hypothesis is confirmed, will confer an anatomic alternative to difficult coracoid infraclavicular brachial blocks or could even become a standard for brachial plexus anaesthesia.

**Trial registration:**

ClinicalTrials.gov, NCT02913625. Registered on 12 September 2016.

**Electronic supplementary material:**

The online version of this article (doi:10.1186/s13063-017-2086-1) contains supplementary material, which is available to authorized users.

## Background

Regional anaesthesia (RA), or the action of blocking nerve conduction temporarily, has been conducted since the beginning of surgical interventions described on humans. With time, techniques evolve, become safer, easier to perform and more effective. When compared to general anaesthesia, RA may offer better outcomes. RA has been linked with reduced postoperative pain, reduced need of postoperative opioids and reduced recovery time in patients undergoing orthopaedic surgery [1, 2]. Thus, when it comes to limb surgery, regional anaesthesia is a very interesting approach.

Simplicity, rapidity and safety of ultrasound-guided (USG) techniques, have propelled the interscalene, supraclavicular, infraclavicular (coracoid) and axillary block as the most popular techniques for shoulder surgery and upper limb surgery [3–6]. Many other approaches have previously been described for upper limb anaesthesia, but their popularity decreased when simpler and safer techniques were reported.

When it comes to elbow and distal upper limb surgery, the infraclavicular approach (ICB) offers many advantages. A Cochrane review reports that coracoid ICB offers a lower likelihood of tourniquet pain, more reliable blockade of the musculocutaneous nerve when compared to a single-injection axillary block, and significantly shorter block performance time compared to multi-injection axillary and mid-humeral blocks [3]. Also, coracoid ICB has been shown to be the easiest way to provide anaesthesia for the brachial plexus [7] because positioning of the operative limb is less painful, the coracoid process is easy to locate and the technique is easy to learn and master [6]. Furthermore, ICB is recommended as the standard of training for anaesthesia residents because of its simplicity and efficiency [7].

Because a steep-angle needle-ultrasound (US) beam is often required, needle visualization remains a challenge in coracoid ICB, particularly when the needle approaches the neurovascular bundle [8, 9]. Because the needle entry point is different, the recently described retroclavicular (RCB) approach offers an almost perpendicular angle between needle and the US beam [10]. This technique has already been described as efficient, rapid, safe and simple to perform in both a recent study [11] and a recent case series [12]. As such, following the Standard protocol items: recommendation for interventional trials (SPIRIT) clinical trial design recommendations (see Additional file 1) [13], our hypothesis is that when comparing RCB with coracoid ICB for upper limb surgery, both techniques will offer similar outcomes except for needle visibility. We believe that the almost perpendicular needle-US beam angle in RCB will provide superior needle visibility to coracoid ICB.

### Objectives

Our general objective is to make a formal comparison between RCB [10, 11] and coracoid ICB for brachial plexus anaesthesia. This study will delineate the differences between the two techniques. Our aim is to compare both techniques in terms of scanning time, needling time, total anaesthesia time, needle visibility, block needle passes, block success and early and late complications. We made the hypothesis that, while providing similar efficacy and better needle visualization than coracoid ICB, the time taken to perform RCB will not exceed the time taken to perform its comparator [3].

## Methods

### Participants, interventions and outcomes

#### Trial design

This study is designed as a prospective, randomized, non-inferiority trial. Two groups of non-consecutive patients will be randomly assigned to either RCB or coracoid ICB. This study will be carried out in two different centres simultaneously.

#### Study setting

The multicentre trial will be conducted in two university hospitals (Centre hospitalier universitaire de Sherbrooke (CHUS) Hôtel-Dieu/Fleurimont in Sherbrooke city and Centre Hospitalier de l’Université Laval [13] in Quebec city).

#### Eligibility criteria

##### Inclusion criteria

Patients eligible for the study must comply with all of the following at randomization:

- Elective or urgent surgery of the hand, wrist, forearm or elbow
- Age >18 years
- American Society of Anesthesiologists (ASA) class I-III
- Able to provide valid written consent
- Minimum body weight of 50 kg, regardless of body mass index (BMI)

##### Exclusion criteria

- Patient refusal
- Previous surgery or gross anatomical deformity of the clavicle
- Systemic or local infection at the needle entry point
- Coagulopathy
- Severe pulmonary condition
- Local anaesthetic allergy
- Pre-existing neurologic symptoms in the ipsilateral limb
- Pregnancy
- Surgical request for an indwelling catheter for postoperative analgesia

Enrolment in another study is not a contraindication as long as both protocols can be applied simultaneously.

#### Interventions

Blocks will be performed by anaesthesiologists experienced in US-guided regional anaesthesia or by PGY-2 to PGY-5 residents under direct supervision by an experienced anaesthesiologist. Operators will have registered a minimum of 25 USG blocks before participating in the study.

Upon arrival in the induction room, standard ASA monitoring will be installed and an 18-gauge or 20-gauge intravenous catheter will be inserted in the contralateral arm. Premedication will be given based on patient preference and the anaesthesiologist’s judgment. Patients will be placed in the supine position with the torso elevated to about 30 degrees and the head facing the opposite side. After a prescan and US parameter optimization, the US probe will then be applied using a sterile technique. A high-frequency linear US probe (6–15 Hz Philips HD11XE Ultrasound; Philips Medical Systems, Bothell, WA, USA, 9–15 Hz Logiq P6; GE Healthcare, Mississauga, ON, Canada, or 15–6 MHz linear probe Sonosite Edge HFL50x; Sonosite Canada inc. Markham, Ontario, Canada) will be used and placed parasagittally just medial to the coracoid process and caudal to the clavicle. Short-axis visualization of the axillary vessels and of the cords will be obtained; the lung and second rib will be identified.

For the retroclavicular approach, the needle insertion point will then be found by palpating the supraclavicular fossa, just medial to the shoulder, at a point sufficiently posterior to the clavicle (generally 1–2 cm depending on morphology and the extent to which the supraclavicular fossa can be depressed) and medial to the trapezius muscle insertion point on the clavicle. This landmark is essential, as the needle will need to clear the clavicle and stay parallel to the US probe to avoid any posterior angling. From this finger position, probe rotation and alignment with the palpating finger will be perfectly achieved while keeping the axillary vessels in the short axis. Once probe rotation achieves finger-probe alignment, the needle will now aim more towards the anterior axillary line than with the coracoid ICB. For optimal imaging and technique the final probe position should rest in the delto-pectoral groove.

For coracoid ICB, the US probe will be applied in the infraclavicular fossa until good visualization of the axillary vessels is obtained and if possible, the brachial plexus cords, then the optimal approach angle and puncture point will be planned.

#### Needling and injection

Under sterile conditions a skin wheal will be made using a 30-gauge needle, and 2 ml of 2% xylocaïne will be applied either behind (RCB) or below (coracoid ICB) the clavicle. A 100-mm 18-gauge echogenic needle, bevel facing up (Plexolong Nanoline; Pajunk Medizintechnik, Geisingen, Germany) will then be inserted into the skin wheal and advanced strictly in-plane with the US beam.

For RCB the needle will cross an initial blind zone measuring 3–4 cm, which corresponds to the acoustic shadow of the clavicle. For safety purposes, this length should be evaluated by checking the surface distance between the probe and initial insertion point. Once the blind zone is crossed, correct in-plane alignment should enable a precise view of the needle shaft and the tip as it advances towards the neurovascular bundle. If the initial puncture point is correct and the angle of penetration is parallel to the US probe, the shaft should point to the posterior wall of the axillary artery. Most importantly, the needle should clear the posterior wall of the clavicle and must not be angled posteriorly, to avoid any risk of pneumothorax.

For coracoid ICB, the needle will be inserted immediately under the clavicle, through the skin wheal, as established previously. The needle will be angled and advanced while aiming for the posterior wall of the axillary artery.

Hydrolocalization with 5% dextrose and neurostimulation are allowed if deemed necessary. Once the block needle tip is located adjacent to the axillary artery and posterior cord, hydrodissection using a local anaesthesia (LA) bolus will enable safe and efficient spread of LA to initially achieve the double-bubble sign [14].

Mepivacaine 1.5% (20 ml) and ropivacaine 0.5% (20 ml) will be injected in a fractionated way, while slightly adjusting the needle tip to obtain a U-shaped distribution of local anaesthetic around the axillary artery.

#### Intervention – concomitant care

Strict adherence to protocol will be mandatory to allow inclusion of patients. Thus, if any other needle or any other local anaesthetics or other volume/dose of local anaesthetics are used, the patient will be excluded from the analysis.

If the patient received more than 2 mg of midazolam (or equivalent dose of other sedative drug) during the procedure, the patient will be included only if he is still able to adequately collaborate during the evaluation of motor and sensory blockade.

This means that patients maintain adequate response to verbal commands and remain competent after the medication administration. In any circumstances, if the drugs given exceed the allowed sedation as mentioned in this protocol (fentanyl 1 μg/kg, propofol 50 μg/kg/min), the plexus block will be considered a failure.

#### Outcomes

##### Primary outcome measure

The primary outcome is the performance time, expressed in minutes. The performance time corresponds to the sum of imaging time and needling time, which both are secondary outcomes. For more details on those times, refer to the secondary outcomes.

##### Secondary outcome measures

- Imaging time: will be measured in minutes and corresponds to the time interval between contact of the US probe with the patient skin and the acquisition of a satisfactory image.
- Needling time: will also be measured in minutes, from the start of the skin wheal to the removal of the block needle from the tissues.
- Sensory loss at 10, 20 and 30 minutes: will be assessed in the territory of the radial (lateral aspect of the dorsum of the hand), median (volar aspect of the index), ulnar (volar aspect of the fifth finger), musculocutaneous (lateral aspect of the forearm), and medial cutaneous nerve of the forearm (medial aspect of the forearm) distributions using a 3-point score, where 0 = normal sensation, 1 = diminished sensation to pinpricks (hypoesthesia), and 2 = loss of sensation to pinpricks (analgesia). The sum of five scores on a maximum of 10 will be the sensory-loss final score. An independent, blinded, research assistant will complete the sensory assessment at 10, 20 and 30 minutes.
- Motor block at 10, 20 and 30 minutes: motor function will be tested (0 = normal strength, 1 = weakness, 2 = paralysis) for the radial (wrist extension), median (thumb-fifth finger opposition), ulnar (fifth finger abduction), and musculocutaneous (elbow flexion) nerves. The sum of the four scores on a maximum of 8 will be the motor block final score. An independent, blinded, research assistant will complete the motor assessment at 10, 20 and 30 minutes.
- Success of plexus block: success is a dichotomic variable answered by yes or no. Success is defined as the completion of surgery without the need for additional LA infiltration, intravenous narcotics, or general anaesthesia. However, light sedation is allowed if deemed necessary by the anaesthesiologist. Light sedation includes midazolam 1 to 4 mg intravenously, fentanyl up to 1mcg/kg. A minimum sensory score of 9/10 will be necessary to proceed to surgery without additional LA infiltration. Patients with an overall sensory score under 9/10 at 30 min will be offered general anaesthesia or supplemental blocks.
- Total anaesthesia time: is measured in minutes and defined as the sum of performance time and time to achieve a minimum sensory score of 9/10. It is the time for readiness for surgery.
- Number of block needle passes: is defined as a unit of 1, 2, 3, etc., and is the number of times the block needle will have to be realigned on the skin in order to achieve its final positioning goal under the axillary artery.
- Needle visualization: procedures will be videotaped and reviewed simultaneously after study completion by 2 independent anaesthesiologists skilled in US-guided regional anaesthesia using a 5-point Likert scale to rate needle visibility (1 = very poor, 2 = poor, 3 = fair, 4 = good, 5 = very good). Needle visibility will be evaluated twice. First, for RCB, assessment will be made when the needle tip is seen 1 cm after crossing the clavicle acoustic shadowing. For the coracoid ICB, the first assessment will be at a needle tip depth of 1 cm. The second needle visibility assessment will be immediately before the local anaesthetic injection, when the visibility is theoretically optimized.
- Needle angle: using the same videotape that we used for the evaluation of the needle visibility, we will note the angle between the needle and the upper side of the ultrasound image. It will be a continuous outcome ranging from 0 to 90 degrees.
- Neurostimulation use: neurostimulation is accepted if needed. However, its use other than for a safety sentinel (defined by <0.3 mA) will be recorded for subsequent analysis. It will be a dichotomous outcome.
- Pain during the procedure: immediately after block completion, patients will be asked to rate their discomfort associated with the procedure using a 10-cm visual analogue scale (0 = no pain, 10 = worst pain imaginable) by an independent and blinded outcome assessor.
- Early and late complications: the incidence of needle-induced paresthesia, vascular puncture, Horner syndrome, dyspnea, and symptoms of LA toxicity will be noted. All patients will be contacted 48 hours after surgery to ask for any delayed complications, such as dyspnea, paresthesia, weaknesses, pain at the puncture site or haematoma. Patients with complications will be followed up by our research group and a consultation in neurology may be sought.

#### Participant timeline

**Fig. 1:**
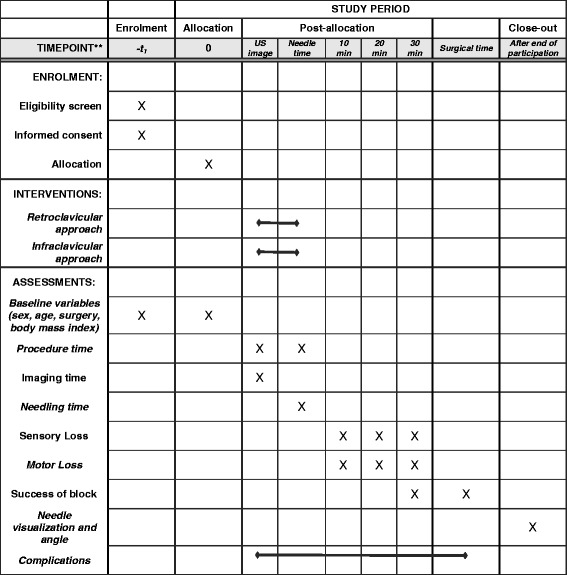
Template of the schedule of enrolment, interventions and assessments. Summary of the study period for each participant, from the recruitment to the close-out

A research member will screen the operating list for any eligible patients. When identified, the patient will first be encountered for the presentation of the research project and possible recruitment. This first encounter will be at least one hour before the scheduled surgery time and will be in the surgical day care unit or in the unit in which the patient is hospitalized. When consent is obtained, patients will first complete a demographic questionnaire. They will then be transported to the induction room for the performance of the plexus block, as previously described. Once the block is completed, patients will remain monitored in a suitable area with appropriate surveillance (induction room, operating room (OR), recovery room) until surgery. The independent research assistant will assess the sensory and motor loss at 10, 20 and 30 minutes (see Fig. 1).

#### Sample size

We decided to conduct a non-inferiority trial with the aim of demonstrating that performance time for the RCB approach is no longer than for the coracoid ICB approach. On the basis of a recent study [15], we established that the performance time for the coracoid ICB approach is 5 min 36 s, with 45 s of visualization time. On the basis of the feasibility trial recently conducted, needling time for the RCB approach is 3 min 42 s [11]. If we use the same imaging time of 45 s, we expect a performance time of 4 min 29 sec for the experimental approach. The standard deviation used for both groups was 2 min 18 s, as reported in the literature [15]. We deemed that a time superiority of 5% would be significant. In other words, RCB performance time will be deemed non-inferior to ICB if the upper margin of the 95% confidence interval created around RCB performance time is under the ICB performance time plus 5%. When using these hypotheses to conduct a non-inferiority analysis, a calculated sample size of 49 patients per group will be required to provide statistical power of 0.90 and one-sided type I error of 0.05. To compensate for potential dropouts or inadequate procedures, we will aim for a sample size of 55 patients per group.

#### Recruitment

As most of the patients will not have been seen previously at the preoperative clinic, we will recruit the patients on the same day as they undergo surgery. The patients will first meet the research team on the surgical day care unit or on the unit where the patient is waiting for surgery. A member of the research team or the attending anaesthesiologist will see the patient at least one hour before the planned surgery schedule and will explain the research project. The patient will receive the consent form at least one hour before surgery and will be given time to read, assess and ask questions before deciding whether or not to participate. The patient will be assured that the quality of care and professionalism will not be affected by refusal to participate.

## Methods

### Assignment of interventions

#### Allocation: sequence generation

Participants will be randomly assigned to either the control or experimental group with a 1:1 allocation ratio as per a computer-generated randomization schedule stratified by sites using permuted blocks of random sizes. The block sizes will not be disclosed, to ensure concealment. Stratification by site will be of particular interest because in this current pre-trial state, one centre mostly uses the retroclavicular approach while the second one uses the infraclaivuclar approach.

#### Allocation concealment mechanism

Sequentially numbered, opaque, sealed envelopes will be used to ensure concealment until enrolment is confirmed. These envelopes will be kept in the locked research assistant office and the recruiting members will have to contact this research assistant to randomize the patient. When the numbered envelope is opened, the patient is automatically included in the analysis and the numbers on each envelope ensure that all patients are enrolled after being randomized. A recruitment log will be created and exclusion will be justified for each patient excluded.

#### Blinding (masking)

In this trial, only the outcome assessor for the motor block and sensory block will be blinded. For obvious reasons, it is not possible to blind the anaesthesiologist performing the block. Moreover, the current setup at the included hospitals does not allow the blinding of the anaesthesiologist supervising the surgery. Thus, the anaesthesia team in the case of block failure will not be blinded. Patients will not be blinded either, as we opted not to fake a plexus block with another needle entry point for organizational reasons and to avoid patient discomfort. However, the primary outcome of performance time should not be affected by patient blinding. Considering that the anaesthesiologist will not be blinded, we did not think it necessary to blind the statistician as, even if blinded, the risk of contamination would be too high.

Discomfort during the procedure and the quality of the motor block and sensory block at 10, 20 and 30 minutes will be assessed by a blinded investigator. Another anaesthesiologist, resident or respiratory therapist will be in charge of evaluating sensory and motor function with pre-established indications to ensure competency. To reinforce blinding, chlorhexidine will be applied over the two theoretical needle entry points and two bandages will be applied.

## Methods

### Data collection, management and analysis

#### Data collection methods

De-nominalized demographic data will be collected by an independent investigator. During the procedure, a standardized data collection document will be used to ensure that all data points are noted and are available for future statistical analysis. For every plexus block, at least two people (operator plus research assistant) will be required for data collection and the research assistant will be responsible for time measurement using a chronometer. Because patient follow up is maintained only up to 48 hours after the surgery, patient retention is not expected to be an issue. Demographic data will include a phone number to allow communication after discharge from the hospital. A presentation to the anaesthesia department and peripheral participating centres will be done to explain the project and pertinent documents.

#### Data management

The patient file number and any additional data allowing patient recognition will be gathered in a different and unique Microsoft Excel file. Every patient will be attributed a number that will correspond to the number in the other data file. These personal data will be on a different locked computer, kept in a locked room at the hospital. These data will be directly transferred from the hand-written form to the locked computer by a research member and will never transit by mail or the Internet. Once the sensitive data are entered onto the computer, any hand-written form allowing participant recognition will be destroyed to avoid the data being divulged. Only research members will have access to the computerized data.

Non-sensitive data will be kept in an Excel folder with a number corresponding to the sensitive data number. A research member will transfer the collected data from the hand-written document to the computerized database. The hand-written document will be kept in case of future need. Back-up data will be kept on the personal computers of the main authors to allow statistical analysis. No related processes to promote data quality will be used, as only research members will be responsible for data transfer from the hand-written document to the computer database. Blocks will be videotaped by recording the screen of the ultrasound device. Patients will not be recorded during these procedures and there will not be any possibility of recognizing the person undergoing the block. Data will be shared by the authors via a password-secured file (Dropbox®) that is only accessible to authors of the study. As soon as the videos are shared between recruiting centres, the videos will be stored in a separate file on the same locked computer as used to store the rest of the information collected.

#### Statistical methods

Analysis of the primary outcome: performance time will be analyzed with a non-inferiority test of the averages, with the objective of finding that the experimental RCB approach takes no longer to perform than the coracoid ICB approach.

The secondary outcomes will all be analyzed using superiority analysis. For continuous data or ordinal data with >8 categories, data will be compiled as average and standard deviation. If data are parametric, Student’s *t* test will be used and if not, the Mann-Whitney test will be used. For dichotomous data (block success, use of neurostimulation), the chi square or Fisher exact test will be used if n >5 or is not >5, respectively. Finally, for ordinal data the chi square test will be used if data are parametric, otherwise the Mann-Whitney test will be used.

Subgroup analysis will be conducted to evaluate if higher BMI influences the outcomes of performance time, needle visibility, number of needle passes and needle angle. Patients will be divided in two groups (of those with higher and those with lower BMI than the average BMI of all recruited patients) and analyzed according to their subgroup. If data are missing or if any patients drop out of the study, data will be analyzed using the intention-to-treat principle.

## Discussion

### Data monitoring

Considering the recent feasibility study that did not identify any significant complications with the RCB approach, which had a similar success rate compared with the coracoid ICB approach, and considering the relatively small number of patients per group, we will not have a formal data monitoring committee. For the same reason, no interim analysis will be conducted.

### Risk of bias

Unfortunately, the proposed methodology implies certain bias that would be hard to eliminate with the current research setup available in the participating centres. First of all, we will have selection bias because we will not be able to approach a consecutive series of patients, as personal resources are not sufficiently available, especially during the on-call hours, when recruitment depends on the anaesthesiologist and their “free” time for recruitment. Moreover, we will have reference centre bias because more anaesthesia providers are competent with RCB in Sherbrooke, and most of the enrolment will be in this university hospital.

For data collection, as it is not possible to double blind this trial for reasons previously described, so we will have performance bias. We might also include attention bias when asking patients if they have discomfort immediately after the plexus block and at 48 hours. However, this approach will be similar and neither group should influence one group more than the other. Finally, we might have therapeutic personality bias as in the consent form we mention our expectation of a shorter and less painful block with the RCB approach. However, we deemed it necessary to mention those facts because they are some of the implied reasons for the implementation of this trial.

For data analysis, we have two principal biases. First, we might have an “all is well in the literature” bias when using the Likert scale to evaluate needle visibility. This scale has been previously used in the similar studies; however, it has never been validated and results should thus be interpreted with caution. Unfortunately, no validated tools exist for this purpose. Furthermore, there might be detection bias even if the outcome assessor for the motor and sensory block is blinded to the procedure. To minimize this we will extend the chlorhexidine application to the two needle insertion points and two bandages will be applied even if no needle was inserted for one of them. However, blood leaking or soaking the bandage might inform the outcome assessor on the approach used.

### Harms

Potential harms are mentioned to the patient as standard before any regional anaesthesia, including risk of pneumothorax, transient or permanent nerve injury, vascular puncture, infections, Horner syndrome or failed plexus block. This practice will be maintained throughout this trial. Any adverse effects or complications will be compiled in the data collection form, but we do not expect to have enough power to find any significant differences. In the feasibility study, no significant or permanent harms were inflicted on the patients with the experimental method. Moreover, we expect better visibility with the RCB approach, which could be associated with reduced risk of complications.

If important harms or concerns about patient safety are noted, we will report to the research group and ethics committee for evaluation on the continuation of the trial. We will also act accordingly in seeking appropriate medical attention if deemed necessary.

### Auditing

No auditing is planned. If considered necessary by the research members, auditing on the quality of the data gathering might be conducted for the specific anaesthesiologist with whom atypical or incomplete data are obtained.

### Confidentiality

Confidentiality is a priority in this project to ensure non-maleficence. Every patient will have a corresponding number. This corresponding number will be linked to the patient file number in an Excel file that will be kept on a locked computer in a locked room within the hospital. No other computer will contain the corresponding numbers with the file numbers. Patient file number and the corresponding number will be inscribed directly on the locked computer and will never transit via the Internet or email. Only the research members may access the locked computer.

Data collected will be in another Excel file that will contain only the corresponding number. No computer will ever have both the Excel files. Thus, we will ensure that no computer loss will allow patient identity to be associated with the collected data. Only the research members and statisticians may have access to the collected data. Videos of the ultrasound machine screen will be sent to the principal investigator centre via a password-protected Dropbox® file and they will be stored in the locked computer with the rest of the patient information as soon as possible. Videos will only contain the ultrasound images and it will not be possible to recognize patients by watching those videos. The name of the video will be the patient number.

## Trial status

At the time of manuscript submission, the recruitment of the patients has started and is ongoing. We have recruited one fourth of the calculated sample size.

## Additional files

Additional file 1: SPIRIT 2013 checklist of recommended items to address in a clinical trial protocol. Corresponds to the checklist of required items in a protocol and the corresponding pages in this manuscript. (DOC 121 kb)

## Abbreviations

BMI: Body mass index
ICB: Infraclavicular
OR: Operating room
RA: Regional anaesthesia
RCB: Retroclavicular
USG: Ultrasound-guided
